# Perceived factors influencing menstrual hygiene management among adolescent girls: a qualitative study in the West Gonja Municipality of the Savannah Region, Ghana

**DOI:** 10.11604/pamj.2022.41.146.33492

**Published:** 2022-02-18

**Authors:** Mubarick Nungbaso Asumah, Abdulai Abubakari, Gifty Apiung Aninanya, Waliu Jawula Salisu

**Affiliations:** 1 Ghana Health Service, Kintampo Municipal Hospital, Kintampo Bono East, Ghana,; 2 Department of Global and International Health, School of Public Health, University for Development Studies, Tamale Northern Region, Ghana,; 3 Department of Health Services Policy, Planning, Management and Economics, School of Public Health, University for Development Studies, Tamale Northern Region, Ghana,; 4 Cambridge Liver Unit, Cambridge University Hospitals NHS Foundation Trust, Addenbrookes Hospital, Cambridge, United Kingdom

**Keywords:** Adolescent girls, Ghana, habits, menstrual hygiene management, menstruation, practices

## Abstract

**Introduction:**

menstrual hygiene (MH) is important for all women, yet it is still a neglected issue in many parts of the world. In most traditional African contexts, including Ghana, menstruation is largely treated as a taboo and humiliating topic that is rarely discussed openly. The main aim of this study is to assess perceived factors influencing menstrual hygiene management among adolescent girls in Ghana´s Savannah Region, West Gonja Municipality.

**Methods:**

we conducted a descriptive exploratory qualitative study among adolescents who had reached menarche. Purposive sampling was used to conduct 24 interviews with 18 teenagers and 6 mothers. Data were analyzed using the thematic content analysis.

**Results:**

the majority (55.5%) of respondents were ≥15 years with maximum and minimum ages being 19 and 13 years respectively. The mean age was 15.7, with a standard deviation of 1.8. A higher proportion (38.9%) of respondents were in their final year (JHS 3). Before menarche, all adolescent girls had heard about menstruation, mostly from their mothers, then from instructors and friends. During menstruation, all of the girls in this study used absorbent products. The cost, comfort, heaviness of menstrual flow, and accessibility influenced the choice of absorbent material, with some respondents utilizing multiple absorbent materials. Some girls were forced to dry their reusable absorbent material in their room because of shyness. Girls thought that reusing absorbent materials after drying them in the sun would have killed germs and removed unpleasant odours. During menstruation, girls are barred from participating in social and religious activities.

**Conclusion:**

culture and religion have very dire consequences on effective menstrual hygiene management. There is therefore urgent need to dispel the growing myths and misconception on menstrual hygiene as well as providing support for adolescent girls for practicing good menstrual hygiene.

## Introduction

Menstruation is a common and natural experience for females during their reproductive years, accompanied by significant physiological and emotional changes [1,2]. Menarche is regarded as a one-of-a-kind sign indicating a girl has attained puberty [3]. According to the Joint Monitoring Programme (JMP) for Water Supply and Sanitation, effective menstrual hygiene management (MHM) entails *“women and adolescent girls using a clean menstrual management material to absorb or collect blood that can be changed in privacy as often as necessary for the duration of the menstruation period, using soap and water for washing the body as required, and having access to facilities to dispose of used menstrual management materials”* [4]. Menstrual hygiene (MH) is essential for all women, yet it is still a neglected issue in most parts of the world [5]. Like Ghana, most traditional African cultures consider menstruation as a taboo and humiliating topic that is rarely discussed publicly [6]. As a result, most adolescent females, particularly in developing countries, are unaware of the need for healthy MHM [7].

Girls are forced to face fear, humiliation, and guilt during menstruation due to a lack of prior information about menstruation [8]. Stillbirths, miscarriages, infertility difficulties, and cervical cancer have been linked to ineffective menstrual management in adolescent girls [8]. Furthermore, research has suggested that infections of the urinary and reproductive tracts are linked to poor MHM in females [9]. In addition, poor MHM may impact girls´ academic performance or lead to school dropout [10]. In developing countries, including Ghana, adolescent girls practice poor menstrual hygiene management. These girls are unable to obtain or purchase sanitary materials to manage their menstrual flow and so they rely on substandard products such as fabric, cotton wool, toilet paper, among others [11-13]. Adolescent girls, in particular those living in rural areas of Sub-Saharan Africa, have serious issues with menstruation hygiene [14]. Different studies have demonstrated that adolescents in underdeveloped countries practice poor MH. Multiple studies conducted in India [15-17] have showed that poor MH ranges from 44.8 to 81.7% among girls. For instance, Poor MH has been reported as 27.0% -40.5% in Nepalese adolescent girls [18,19], in Bangladesh, 68.5% of girls practiced poor MH [20]. The prevalence of poor MH in Uganda is 74.7% [18], 10.0% in Egypt, [19], 28.8% to 35.7% in Kenya [20,21] and between 9% to 76% among adolescent girls in Ethiopia [22-24].

Furthermore, studies conducted in Ghana have showed that the prevalence of poor MH ranges from 19.8% to 68.9% among adolescent girls [13,14,25,26]. Studies on menstrual hygiene management among female adolescents were conducted quantitatively. There are woefully scares qualitative studies on MHM in Ghana. Despite these studies, there is limited literature on perceived factors influencing MHM among adolescent girls in the newly Savannah Region. This study therefore aims to assess the perceived factors influencing menstrual hygiene management among adolescent girls in the West Gonja Municipality of Savannah Region of Ghana. As a new region, studies like these are needed to inform policy decision in the areas of girl child education, health and sanitation.

## Methods

**Study setting:** this study was conducted in the West Gonja Municipality of the Savannah Region of Ghana. Damongo is the capital of the municipality as well as the regional capital. The study setting was chosen because no study on this topic was conducted in the area. As a new region, these studies are essential to the policy making in the areas of sanitation, water and girl child education.

**Study design:** descriptive exploratory design using qualitative approach was used.

**Sample population:** the study included adolescent girls who had had their first menses. These girls were enrolled because they had had at least a menstrual cycle and so could provide relevant information based on their respective experiences.

**Sample size and sampling technique:** participants could speak English or Gonja. A purposive sampling technique was used in this study to select participants. The researchers recruited eighteen (18) adolescents for face-to-face individual interviews. Also, six (6) mothers were recruited into the study to provide in-depth information on MH to clarify and support information adolescents provided. The sample size was determined when saturation was reached.

**Data collection tool and procedure:** the interview guide was developed after reviewing similar studies. The interview guide was structured in relation to the study objective. The interview guide was pre-tested in three (3) adolescents and one (1) mother. The interview guide was also shared with senior colleagues with considerable knowledge on the subject to do a face validation. All comments arising from peer validation and pre-testing were addressed before the data collection commenced. The Interview guide for both adolescent girls and mothers included questions on socio demographics characteristics, knowledge on menstruation, practice of MHM and factors influencing MHM. All questions were open ended. This allowed researchers to probe to unravel all issues. Participants who had had their first menses were introduced to the researchers. Participants were given the option of indicating date, time, and location of their interviews. The majority of the interviews took place in the participants´ homes, with a few of them taking place in school. In all, 24 interviews were conducted, comprising 18 adolescents and 6 mothers. The interviews were performed in either English or Gonja, with an interview guide based on the study´s goals. The participants gave their permission for the interviews to be recorded. The interviews in English were transcribed verbatim, whereas the interviews in Gonja were transcribed based on their meaning. A Gonja language specialist was asked to authenticate transcripts that were in Gonja, and the procedure was kept discreet. The interviews ranged from 20 to 35 minutes in length.

**Data analysis:** a manual thematic content analysis was used to analyse the data. Audio recordings were transcribed verbatim. Transcribed data were double-checked for correctness. Researchers coded transcribed material separately before having many discussions to produce themes and subthemes.

**Ethical Consideration:** the ethical approval was obtained from the Committee on Human Research, Publications & Ethics (CHRPE) with a reference number (CHRPE/AP/199/20). Also, permission was granted by the District Education Directorate for the study to be carried out. Parents or guardians of all participants had signed written consent and all participant (less than 18 years) assented to participate in the study. Respondents had the liberty to withdraw from the study at any point in time. Anonymity was maintained by representing participants with numbers.

## Results

**Demographics of adolescent girls and mothers:** Table 1 below shows the socio demographic characteristics of adolescent girls recruited for this study. The majority (55.5%) of respondents were aged 15 years or above with maximum and minimum ages being 19 and 13 years respectively. Mean age was 15.7 with a standard deviation of 1.8. A higher proportion (38.9%) of respondent were in their final year (JHS 3) and 44.4% being Gonjas. The majority (55.6%) of the participants were Muslims. Table 2 shows the demographic characteristics of mothers. A total of six (6) mothers with a minimum age of 28 and maximum age of 47 were interviewed. Two (2) mothers were traders, another two (2) were housewives, then there was a teacher (1) and a nurse (1). The respondents were predominately (five out of six) Muslims and half were Gonjas.

**Table 1:** socio-demographic characteristics of adolescent girls

Variables	Categories	Frequency (N=18)	Percentage (%)
**Age**	<15 years	8	44.4
	≥15 years	11	54.6
	Mean (SD)		15.7±(1.8)
	Minimum age		13 years
	Maximum age		19 years
**Class of students**	Form 1	5	27.8
	Form 2	6	33.3
	Form 3	7	38.9
**Residence**	Urban	9	50
	Rural	9	50
**Ethnicity**	Gonja	8	44.4
	Dagarti	4	22.2
	Frafra	1	5.6
	Kamara	3	16.7
	Hanga	1	5.6
	Sissala	1	5.6
**Religion**	Christian	8	44.4
	Muslim	10	55.6

**Table 2:** demographic characteristics of mothers

Pseudo Name	Age	Occupation	Number of children/dependences	Residence	Ethnicity	Religion
Mother_1	30	Teacher	2	Urban	Gonja	Muslim
Mother_2	40	Trader	7	Rural	Dagarti	Christian
Mother_3	47	Housewife	5	Rural	Gonja	Muslim
Mother_4	28	Trader	1	Urban	Kamara	Muslim
Mother_5	34	Housewife	3	Rural	Hanga	Muslim
Mother_6	27	Nurse	1	Urban	Gonja	Muslim

### Knowledge of menstruation and menstrual hygiene

#### Information prior to menarche

Almost all the participants were informed about menstruation prior to their menarche. However, the information given was scanty and so these girls were required to see elderly women in the family when they started to experience their menses. *“…I was told that at a certain point in my life I would see blood from my vagina. I was informed to report to an elderly woman in the house when I see the blood for support …”* (Student_3, 17 years).

### Mode of communication on menstruation

For some participants, fear and calmness was used to communicate about their menstruation. “…She (my mother) told me that now that I have experienced my menses, I am a woman. She cautioned me to abstain from sex. She added that, my friends would have made fun of me and that I might even die in attempting to give birth or abort pregnancy …” (Student_6, 15 years).

### Source of information and challenges

The sources of information on menstruation and hygiene-related issues vary. These sources include; mothers, teachers and friends (peers). Some mothers opined that, some of this information acquired from peers were born out of uninformed talks and full of myths and hearsay. The sources of information on menstruation and hygiene-related issues vary. These sources include; mothers, teachers and friends (peers). Some mothers opined that, some of this information acquired from peers were born out of uninformed talks and full of myths and hearsay. This is what some mothers said: *“…Sometimes, it is difficult because they do not open up on menstruation issues unless they trust you. Usually, they consult their trusted friends for assistance; sometimes, information offered by these friends however is not always accurate…”* (Mother_4, 28 years). Most girls resort to their mothers for information on menstruation before menarche. *“..Before I saw my menses, my mother had given me information on menstruation and how to go about it when I would have seen it…”* (Student 16, 17 years). Most girls had information about menses from their teachers. *“…We were taught in school about menses and how to manage them by our science teacher...”* (Student_18, 15 years).

### Menstrual hygiene practices among adolescent school girls

#### Use of absorbent materials

All participants used absorbent material during menses. The material of choice is dependent on personal preference, experience, merits and demerits of the absorbable materials. The absorbent materials being used are; sanitary pad, cloth, tissue paper and cotton. *“... I use sanitary pad because it makes me feel comfortable and oftentimes nobody knows that I am menstruating…”* (Student_15, 18 years). Two (2) participants also used more than one absorbent material during menses. Student_11 said: *“…Sanitary pads are good but some makes me develop sores (groin areas). That makes me unable to walk well. To reduce the sores, I used both cloths and pad…”* (Student_11, 19 years). The advantages and disadvantages of these absorbent materials differ from person to person, and this influences the choice of these materials. It is revealed that, three (3) participants preferred sanitary pads in spite of the challenges. *“… I prefer to use sanitary pad, but when the flow becomes scanty or little, I resort to the use of tissue paper or cotton. The tissue does not absorb enough blood and starts to tear up and even gets stuck in the vagina …”* (Student_8, 18 years) *“…I prefer using sanitary pad because with the cloth you need to be extra careful if not you can be exposed…”* (Student_4, 16 years). Two mothers believed that both cloth and sanitary pads, which were commonly used among girls, had some disadvantages which put them in a dilemma. *“…Sometimes, some of the sanitary pads are impregnated with medications but I do not know if it has effect on me, it makes you feel very uneasy whilst wearing it. When you wash the cloth, you must be careful that it is clean enough; otherwise, it can harm you. I therefore do not even know which one to use. But most of my children use the sanitary pad and sometimes, the cloth…”* (Mother_3, 47 years).

### Management of menstruation

Some girls are told how to manage their menstrual flow with the use of sanitary pad, cloth or cotton. Two (2) mothers indicated how their daughters managed menstrual flow, as you can read in the following extract: *“…When my children begun to menstruate, they informed me. So, I took them through ways to keep themselves clean. The use of cotton and old cloth are difficult for the first-time users. Now, they are able to use any absorbent material with ease…”* (Mother_2, 40 years).

### Hygiene practices during menses

All study participants bathed during menses. The foul odor and sticky thighs due to menstrual blood were the main reason for bathing among menstruating girls. Nine (9) participants used water only to wash the vagina, three (3) used water and antiseptic to bathe and wash the vagina to drive away the odor. *“…. I sometimes smelled during my menses. I bathed with antiseptic and sweet-scented soap to take away the smell…”* (Student_6, 15 years). Five (5) participants strongly disagreed with the use of antiseptic and soap to wash the vagina as this was associated with some discomfort and illness. *“…. you cannot wash your genitalia with soap and antiseptic. When it enters the vagina, you would feel some unusual pain. I just use water to wash my vagina.”* (Student_17, 16 years). Another participant said: *“… When I use the soap to wash my vagina, I feel very uncomfortable. So, I think it´s better to wash with water only since it takes away the smell as well…”* (Student 9, 19 years). During menses, sometimes the menstrual blood may stick on the thighs. For four (4) participants that made them uncomfortable and to relieve themselves, they bathed frequently. Participants bathed twice daily. *“.. I usually bathe twice. Sometimes, when the menstrual flow is heavy or the absorbent material is not absorbing enough, some of the blood can leak to the thighs. This can be very discomforting unless you bathe…”* (Student_5, 14 years).

### Cleaning and drying of reusable absorbent material

Ten (10) participants held the view that; the cloth needs to be wash with soap and water and dried up of direct sunlight after use. *“…I usually wash my used cloth after bathing. I wash it with soap and water, and dry it of direct sunlight…”* (Student_7, 16 years). Shyness compels five (5) participants to inappropriately wash the cloth and as well dry them inside the room *“.…sometime when you are washing the cloth and people come in. you become shy, and so to avoid the shyness, you would just rinse it and quickly dry it in the room. This makes the cloth to smell. But if air blows on it, the smell is much better…”* (Student_4, 16 years).

### Disposal of used absorbent materials

There are varied ways in which used sanitary pads and other absorbent materials are disposed of. These include; refuse dump/bush, gutter, drains and toilets. The disposal mode is influenced by some cultural beliefs of the participants. *“F I wrap it with rubber and throw it into the bush or put it in dust bin so that it is disposed of with other refuse in the bush where nobody would see it...”* (Student_9, 19 years). *“…I just wrap it with rubber, and then dig a hole and cover it with the earth or dispose it in the toilet...”* (Student_14, 18 years). The reason for the choice of a disposal site has some cultural influence. This is what two (2) mothers said: *“…We were told not to dispose of them in the dust bin or refuse dump because this has traditional ramifications. You may never give birth, if it is seen by a wicked man…”* (Mother_2, 40 years). *“…Is not good to dispose of it in the gutter or refuse dump because a pig or dog could pick it up and send it back to the community. So, in this era of ritual money, someone could use the blood for rituals to become rich…”* (Mother_4, 28 years). Factors affecting menstrual hygiene managementIt was revealed that socio-cultural factors, socio-economic factors, social support, availability of and ability to reuse the absorbent material were factors that immensely influenced the practice of menstrual hygiene.

### Religious and cultural influence on hygiene practice

Religious and cultural practices influence greatly the practice of hygiene. The participants opined that during menstruation, there were some restrictions with respect to the inability to participate in social activities such as cooking, fetching water and others. *“..During menstruation, we are considered impure until the menstrual blood stops and a special bath is performed to make you pure and thereafter you can participate in religious activities…”* (Student_11, 19 years). There are some restrictions that are influenced by the culture of the person(s) including cooking, fetching water and others. All mothers said: *“...In some housing for older people, menstruating women do not fetch fire from that house. It is because the elders may be tabooing that. Growing up, we were told that, you can ‘spoil´ someone´s ‘medicine´ by fetching the fire. We are not allowed to cook for those people whilst in our menses. If you are selling food, you are advised to let someone help you during these times. In some homes too, a menstruating woman is not allowed to sow seeds and even fetch water for domestic use of the family…”* (Mother_1, 30 years).

### Social support

Parents, especially mothers, provide the needed support to the girl during menstruation to enable them effectively manage their MH experiences. *“..For me my mother supports me with a sanitary pad anytime I experience my menstrual flow. My father too is very helpful, he sometimes gives me money to buy my basic needs. I use a part of it to purchase pad for my usage…”* (Student_1, 14 years). *“.. sometimes, my father gives me money to go and buy pads. Also, my mother always buys them for all of us. So anytime we get the money we give it to our mother to buy them or we just buy them and come and leave them in the room for everybody to use…”* (student_13, 15 years).

### Availability of materials

The non-availability of absorbent materials and the distance to getting absorbent materials also hinders their use. Three of the participants made this observation; *“…In our village, nobody sells sanitary pads. So, when you start to menstruate, you are left with no option but to explore other materials including cloth, cotton or tissue paper. To buy them we have to travel a long distance …”* (Student_12, 15 years).

### Cost and reusability of absorbent materials

Two (2) mothers held the view that the cost and reusability of absorbent material influence the choice of absorbent material used during menses. This is what a mother said: *“… I am a widow. We struggle to get our daily bread. I have heard about sanitary pads, but because of the cost, my children use cloth anytime they experience their menstrual flow. The little money I get is used to buy food…”* (Mother_3, 47 years). The ability to reuse this material has a greater influence on the type of material to use. They think that this would save cost. *“…Cloth and cotton are cheap. Moreover, cloth can be used for a longer time. Due to the cost of the sanitary pad, I used it for the first few days of my menses. After two to three days, the menstrual flow became scanty. Now, I use cloth with ease….”* (Student_1,14 years).

## Discussion

The study aims at assessing the perceived factors influencing menstrual hygiene management among adolescents in the West Gonja Municipality of Savannah Region of Ghana. Adolescents´ access to menstrual information and the sources of this information before menarche, almost the respondents received menstrual information before their first period. This finding corroborated to the evidence of Sharma and Gupta [27] in India. In contrast to the above statement, Shah *et al*. [28] found that in rural India, most girls had no information on menses prior to menarche. Cultural and religious dynamics may have contributed to this disparity.

The main sources of this information were parents/guardians, teachers. The majority of girls received information from their parents (mothers). This is in line with the findings of [29,30], who found that mothers were champions in spreading menstrual hygiene and menstrual hygiene knowledge. In some cultures, as demonstrated by Gorah *et al*. [30], it is the responsibility of grandmother or mother to transfer information on menses to their girls. Contrary to the above, Pradeepkumar *et al*. [31] showed that most girls received information on menstruation through their friends. However, mothers in the current study were of the view that information obtained from friends were usually born out of hearsay and usually unscientific. The view of these mothers was supported by other researchers [32,33]. This, therefore, explain why mothers were the preferred source of information on menstruation in this study.

This study also revealed that participants used sanitary pads, old(used) cloth, with a few participants using cotton and tissue as absorbent material. Available literature has shown that the majority of girls used sanitary pads. For instance, 93.8% of respondents used sanitary pads in Nepal [34], and 67.2% in Nigeria [30]. Contrary to the above, in rural Ghana, 47.2% said to be using sanitary pads [13], in Northern Ghana about 20% used sanitary pads [26]. In the present study, the authors could not quantify responses to make a proper comparison. Also, the study participants were only schoolgirls, unlike previous studies where girls in and out of school had been all recruited. Also, the belief of these girls may differ and hence could account for the disparities. Some girls in the study use cloth. A study conducted in India by Pradeepkumar *et al*.[31] showed that the majority of the respondents use cloth as absorbent material. The socio-economic status and socio-cultural factors could be linked to the differences in the findings.

The participants indicated that the absorbent materials were wrapped in plastic bags before being disposed. According to Emmanuel and Yawson [35] and Neupane *et al*. [34] the majority of girls wrapped their used absorbent materials with plastics before disposing them. Most girls in this study disposed their sanitary materials in the toilets. This is consistent with a study in rural Ghana [13] where most respondents used the toilet as the preferred dumping site for their used absorbent materials. This similarity is due to the fact that the study subjects were within similar geographical locations with similar characteristics. Mothers indicated that disposing of used sanitary materials in the open has some spiritual consequences. They averred that a wicked person could take your used pad to make you barren. Also, fraudsters commonly referred to as “Sakawa” boys could also use the blood for rituals which can make you poor for the rest of your life. Interestingly, Emmanuel and Yawson, [35] revealed that most girls disposed their used sanitary pad in the dustbin. Despite sharing the same geographical area, the evident cause of this disparity may be ascribed to resource distribution discrepancies between Ghana's urban and rural areas, as well as cultural variances. In comparison to urban regions of Ghana, dustbins are not frequently utilized in rural Ghana.

Most of those who use reusable absorbent material clean it with water and soap. This is in line with a study conducted in rural Uganda by Hennegan *et al*., [36] where the majority of respondents cleaned their clothes with soap and water. Contrarily, in Mumbai India, most respondents washed their reusable absorbent materials with only water [37]. This disparity might be explained by parents´ financial situation. It´s possible that soap isn´t available for cleaning absorbent fabrics, forcing them to wash them with simply water.

In the current study, some participants dried reusable absorbent materials in the sun. The ideal approach is to dry these materials in the sun since sunlight acts as a natural sterilizer, cleansing the material of any germs that might affect the user´s health [38]. Despite the importance of drying absorbent materials in the sun, several mothers and girls in this study said that shyness and lack of private drying areas forced them to dry their absorbent materials in their rooms. This is corroborated by UNICEF [39] which identified a lack of privacy and stigma, as well as the absence of private venues to wash and dry used absorbent materials as reasons for not drying used absorbent materials outside. In light of the foregoing, Hennegan *et al*. [36] observed that some girls in a Ugandan boarding school were embarrassed to wash their work clothes in public, so they cleaned and dried them in the dormitory at night.

A minority of the respondents used soap and water in washing their genitalia. This was similar to the study undertaken by Belayneh and Mekuriaw [10] which reported that about 70% of participants used soap and water to clean their genitalia during menstruation. In this study, some girls held the view that menstrual blood had a foul smell, and so for some of these girls´ washing genitalia with strong scented soaps and water to take away the smell. A stronger opposition was raised by some respondents that, using soap and water to clean the genitalia has nothing to do with foul smell. They opined that foul smell came about when girls did not treat their reusable absorbent materials well. For some of these girls who disagreed with the earlier assertion, they based their argument on the fact that using soap and water caused irritation of their genitals. Some recalled that they learned in school that washing the genitalia with soap and water could amount to douching. This assertion is supported by a study in Nigeria where some adolescents did not wash their genitalia with soap and water as a result of irritation [40].

Participants opined that during menstruation, there were restrictions with respect to worship as well as the inability to participate in social activities such as cooking, fetching water, and others. Religion and cultural practice influence greatly the practice of menstrual hygiene. There is a vast literature available to demonstrate that culture and religion have an influence on menstrual hygiene management [41-46]. In Nigeria, some adolescents within Bokkos in the plateau state must wash and dry used absorbent materials in secrecy. These women are not allowed to engage in religious activities, and they are not allowed to cook [30]. Also, in Chitwan, Nepal, (92.7%) girls did not partake in religious activities or visit the temple and 74.6% of the girls did not visit the kitchen talk less of cooking whereas some 16.1% of respondents avoided certain food such as banana [34]. In India, menses are considered dirty and polluting, hence most girls experience restrictions on cooking, work activities, sexual intercourse, bathing, worshipping and eating certain foods [38]. This further shows that issues of menstruation are not peculiar to a specific continent. Somehow, every country and continent have some specific way of life that does not allow menstruating women to live a normal life during their period.

**Limitations:** as in qualitative studies, findings in this study are not too generalized. Where generalization is intended, it ought to be done with extreme caution. Also, study subjects were selected purposively as such they might be selection bias.

## Conclusion

Overall, most adolescent girls are making efforts to practice good MHM. However, culture and religion have very dire consequences on effective menstrual hygiene management. There is therefore urgent need to dispel the growing myths and misconceptions on menstrual hygiene as well as provide support for adolescent girls in practising good menstrual hygiene. Formation of adolescent groups by facilitators to provide information on adequate MHM practices is also needed. Quantifying the dangers related to poor menstruation management, school absence and involvement, and risk sexual exposure, as well as developing cost-effective remedies, in-depth quantitative study is required.

### What is known about this topic

- *Menstrual hygiene (MH) is essential for all women, yet it is still a neglected issue in most parts of the world;*
- *Misinformation about menstruation influence poor menstrual hygiene practices*.

### What this study adds

- *The cost, comfort, heaviness of menstrual flow, and accessibility influence the choice of absorbent material;*
- *In Northern Ghana, culture and religion were identified as the key determinants to menstrual hygiene management habits;*
- *Sensitization of adolescent girls on their sexual and reproductive health (SRH) before menarche could lead to adequate MHM practices*.
